# Advances in loading−release−repair strategies of hydrogels for anterior segment disease therapy

**DOI:** 10.3389/fimmu.2026.1897244

**Published:** 2026-07-07

**Authors:** Xiaodi Jiang, Ruijie Xu, Qihui Zhou, Xuelian Yin, Xiaoting Peng, Huixin Zhang, Yaqin Jiang

**Affiliations:** 1 School of Clinical Medicine, Shandong Second Medical University, Weifang, China; 2 Weifang Eye Hospital, National Key Clinical Specialty, Zhengda Guangming Eye Group, Weifang, China; 3 Qingdao Key Laboratory of Smart Rehabilitation Material, Shandong Engineering Research Center for Tissue Rehabilitation Materials and Devices, School of Rehabilitation Sciences and Engineering, University of Health and Rehabilitation Sciences, Qingdao, China; 4 School of Pharmaceutical Sciences, Shandong University, Jinan, China; 5 Weifang Eye Institute, Weifang, China

**Keywords:** anterior segment diseases, drug delivery, hydrogels, intelligent controlled release, tissue repair

## Abstract

Anterior segment (AS) diseases are a major group of ocular disorders that impair ocular surface homeostasis, optical transparency, aqueous humor circulation, and postoperative visual quality, including Dry Eye Disease (DED), corneal injury, Infectious Keratitis (IK), uveitis, glaucoma, and Posterior Capsule Opacification (PCO). The therapeutic goal of these diseases extends beyond symptom relief toward the establishment of integrated treatment systems capable of overcoming complex ocular barriers and actively regulating the pathological immune microenvironment and inflammatory cascades through efficient drug loading, controlled release, and tissue repair. However, conventional ophthalmic formulations such as eye drops and ointments remain limited by tear dilution, blinking clearance, nasolacrimal drainage, corneal epithelial barriers, restriction by the Blood-Aqueous Barrier (BAB), and poor long-term compliance, making it difficult to maintain effective drug concentrations at lesion sites. Hydrogels, composed of hydrated polymeric networks, have emerged as promising platforms for AS disease therapy owing to their excellent biocompatibility, transparency, tunable mechanical properties, tissue adhesiveness, stimuli responsiveness, and extracellular matrix (ECM)-mimicking structures. Depending on clinical requirements, hydrogels can be engineered into adhesive eye drops, thermosensitive *in situ* gels, corneal repair scaffolds, drug-eluting contact lenses, intracapsular drug reservoirs, and functionalized intraocular lenses (IOLs) to prolong ocular retention, enhance trans-barrier delivery, reduce administration frequency, and remodel the pathological microenvironment through anti-inflammatory, immunoregulatory, antioxidative, antibacterial, pro-regenerative, and anti-fibrotic effects. Centered on the “loading−release−repair” principle, this review systematically summarizes material design, functional mechanisms, and application advances of hydrogels in the treatment of prevalent AS diseases, aiming to provide insights for performance optimization, disease−specific adaptation, and clinical translation of next−generation ocular hydrogels with immune-microenvironment targeting capabilities.

## 1 Introduction

The anterior segment (AS) represents a vital anatomical region responsible for sustaining clear vision and intraocular homeostasis, which mainly consists of the tear film, conjunctiva, cornea, anterior chamber, iris, ciliary body, trabecular meshwork, lens, and lens capsular bag, among other structures (1–6). Exposed directly to or indirectly connected with the external environment, this region performs multiple physiological functions, including optical refraction, transparent barrier formation, tear secretion regulation, immune defense, aqueous humor circulation, intraocular pressure (IOP) maintenance, and postoperative tissue repair (3, 7–11). AS disorders such as corneal injury, dry eye disease (DED), infectious keratitis (IK), uveitis, glaucoma, and posterior capsule opacification (PCO) following cataract surgery differ in etiologies and clinical manifestations, yet they commonly share pathological processes including barrier disruption, innate immune activation, inflammatory amplification, oxidative stress, infectious invasion, aberrant cell migration and fibrotic remodeling (12–31), in severe cases, these conditions may trigger corneal opacification, elevated IOP, visual axis obstruction and irreversible visual function impairment (20, 21, 25, 26, 28, 31).

At present, treatments for AS diseases still mainly rely on eye drops, eye ointments, conventional ophthalmic gels, topical anti−inflammatory or anti−infective agents, intraocular pressure−lowering medications, and surgical interventions (1–4, 12, 13, 21, 27, 32). Nevertheless, the unique anatomical and physiological barriers of the eye markedly constrain the therapeutic efficacy of these modalities (1–5, 32). After instillation, eye drops are prone to rapid clearance *via* tear dilution, blinking reflexes, and nasolacrimal drainage, which hinders the formation of a stable drug reservoir at lesion sites (2–4, 32). Tight junctions of the corneal epithelium and the blood-aqueous barrier (BAB) further impede drug penetration into deep ocular tissues, resulting in low concentrations of effective agents reaching target sites (1–4, 32). For chronic disorders including DED and glaucoma, long-term and frequent drug administration tends to reduce patient compliance (12, 13, 25, 27, 33, 34). In cases of corneal injury and IK, monotherapy fails to satisfy multi-stage repair requirements simultaneously, such as anti-infection, anti-inflammation, regeneration promotion, and anti-scarring effects (14–24). As for PCO, neodymium−doped yttrium aluminum garnet (Nd: YAG) laser posterior capsulotomy serves as a passive intervention after disease onset. It cannot block abnormal proliferation, migration, and epithelial−mesenchymal transition (EMT) of residual lens epithelial cells (LECs) in the early postoperative phase (31, 35–39).

Inspired by the shared features of AS diseases, namely high physiological barriers, short ocular retention, and multi−stage pathological repair, and sustained inflammatory immune imbalance, hydrogels have gradually emerged as a pivotal material platform for ophthalmic drug delivery systems and tissue regeneration (3–6, 10, 11, 40, 41). Despite the ease of administration of conventional eye drops, they are susceptible to tear dilution, clearance induced by blinking and nasolacrimal drainage, making it difficult to construct a stable drug reservoir on the ocular surface (2–4, 32). Although delivery systems such as nanoparticles, liposomes, and microspheres can enhance drug loading and sustained−release capacity, their standalone application frequently suffers from insufficient ocular surface retention, local irritation, particle aggregation, or inadequate long−term safety evaluation (2, 10, 32, 42). Drug−coated intraocular lenses or surface−modified biomaterials are capable of inhibiting cell adhesion and proliferation to a certain extent, yet they are limited by low drug loading, as well as imprecise control over release duration and spatial distribution (35–39). While conventional sutures, tissue adhesives, or inert filling materials are available for structural repair, they fail to simultaneously achieve drug delivery, inflammatory modulation, and tissue regeneration (15–19). By contrast, hydrogels possess distinctive properties including three−dimensional hydrophilic networks, high water content, flexible mechanics, favorable biocompatibility, optical transparency, tunable degradability, tissue adhesiveness, and stimuli responsiveness, which are highly compatible with the moist, soft, and transparent microenvironment of the ocular surface and intraocular tissues (3–6, 10, 11, 15–19, 40, 41). Compared with traditional eye drops, hydrogels are able to prolong ocular surface drug retention and reduce administration frequency (3–5, 32, 40). Relative to simple nanocarriers or drug coatings, hydrogels enable synergistic drug loading, dynamic composite construction, stimuli−responsive intelligent release, and local microenvironment regulation through network structures, functional groups, and nano−components (3, 6, 10, 11, 34, 36–39, 41, 43–46). In comparison with conventional surgical filling materials, extracellular matrix (ECM)−mimicking hydrogels can further provide structural support for epithelial migration, stromal remodeling, and scar−free repair (15–19). Accordingly, hydrogels are not merely regarded as sustained−release materials substituting conventional formulations. Instead, they function as an integrated therapeutic platform integrating localized delivery, tissue adaptability, immune microenvironment regulation, and pathological microenvironment remodeling (3–6, 10, 11, 40, 41). In recent years, researchers have developed adhesive and antioxidant hydrogel eye drops for DED (43, 47), basement membrane−mimicking and Janus hydrogels for corneal repair (14–19), sequential antibacterial−regenerative hydrogels for IK, drug−loaded contact lenses and long−acting contact−mediated delivery devices for glaucoma (20–24), as well as intracapsular hydrogel drug reservoirs and functionalized hydrogel−modified intraocular lenses (IOLs) for PCO prevention (35–39).

**Figure 1:**
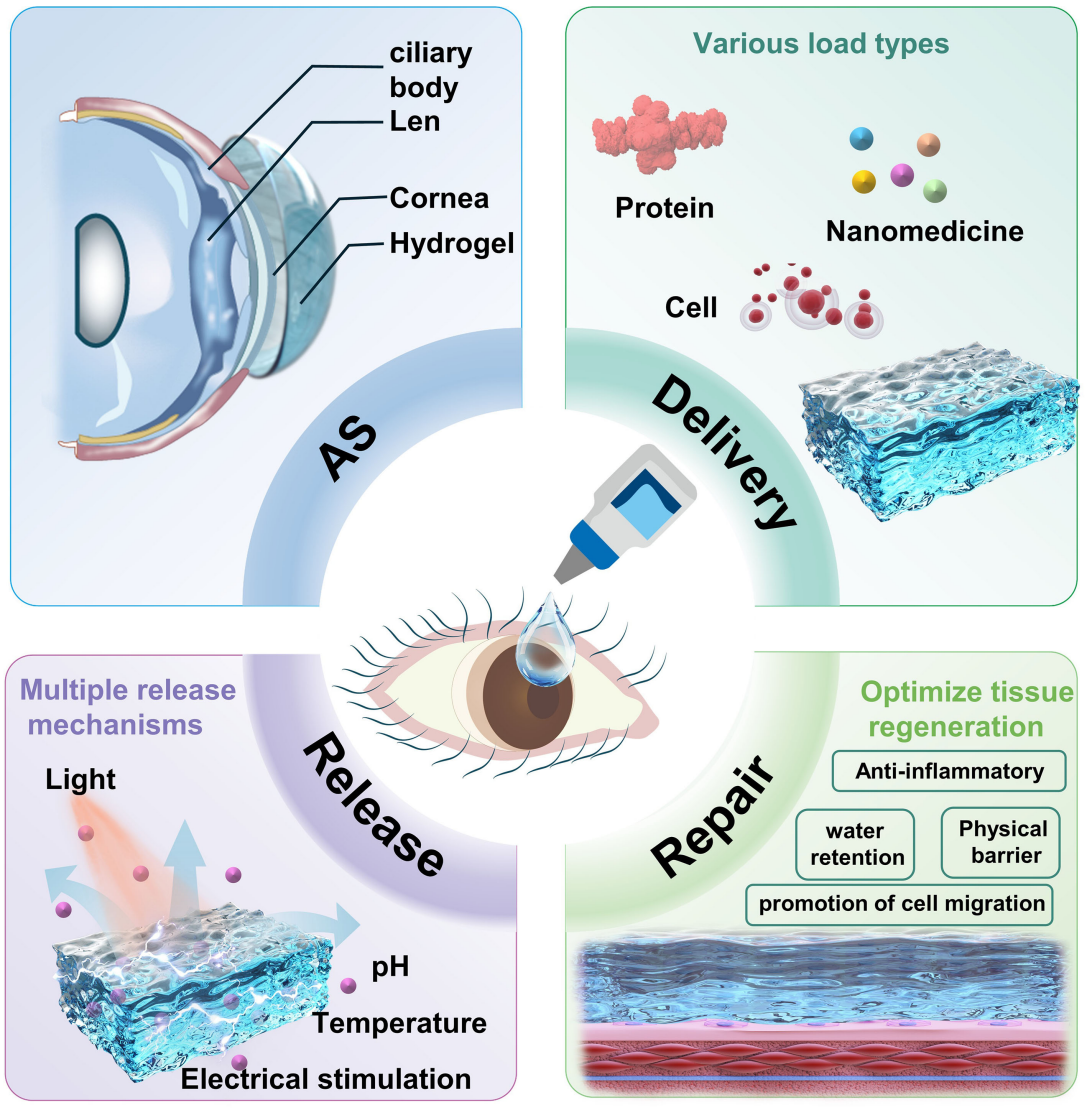
Schematic diagram of design strategies and representative studies of hydrogels in the treatment of common AS diseases.

On this basis, we discuss the potential advantages of hydrogels in the treatment of AS diseases. This paper first elaborates on the pathological mechanisms underlying AS disorders. Centered on the core theme of hydrogels featuring “active ingredient loading, intelligently controlled release, and targeted tissue repair”, we systematically summarize the design strategies and representative research advances of hydrogels for the management of prevalent AS diseases (**Figure 1**). Finally, we conclude the impacts exerted by hydrogels *via* “loading-release-repair” on microenvironment modulation, tissue regeneration, and related fields. Unlike conventional ocular drug-delivery reviews that primarily classify formulations by carrier type or administration route, the present review uses the “loading-release-repair” framework to connect material design with the sequential therapeutic needs of AS diseases. This process-oriented framework is particularly suitable for the AS because successful treatment requires not only local drug retention and controlled exposure, but also restoration of transparent barriers, regulation of inflammation and infection, and prevention of fibrosis or postoperative tissue remodeling.

## 2 Anatomical and physiological characteristics, common pathological features, and mechanisms of AS

### 2.1 Anatomy and physiological functions of the AS

**Figure 2:**
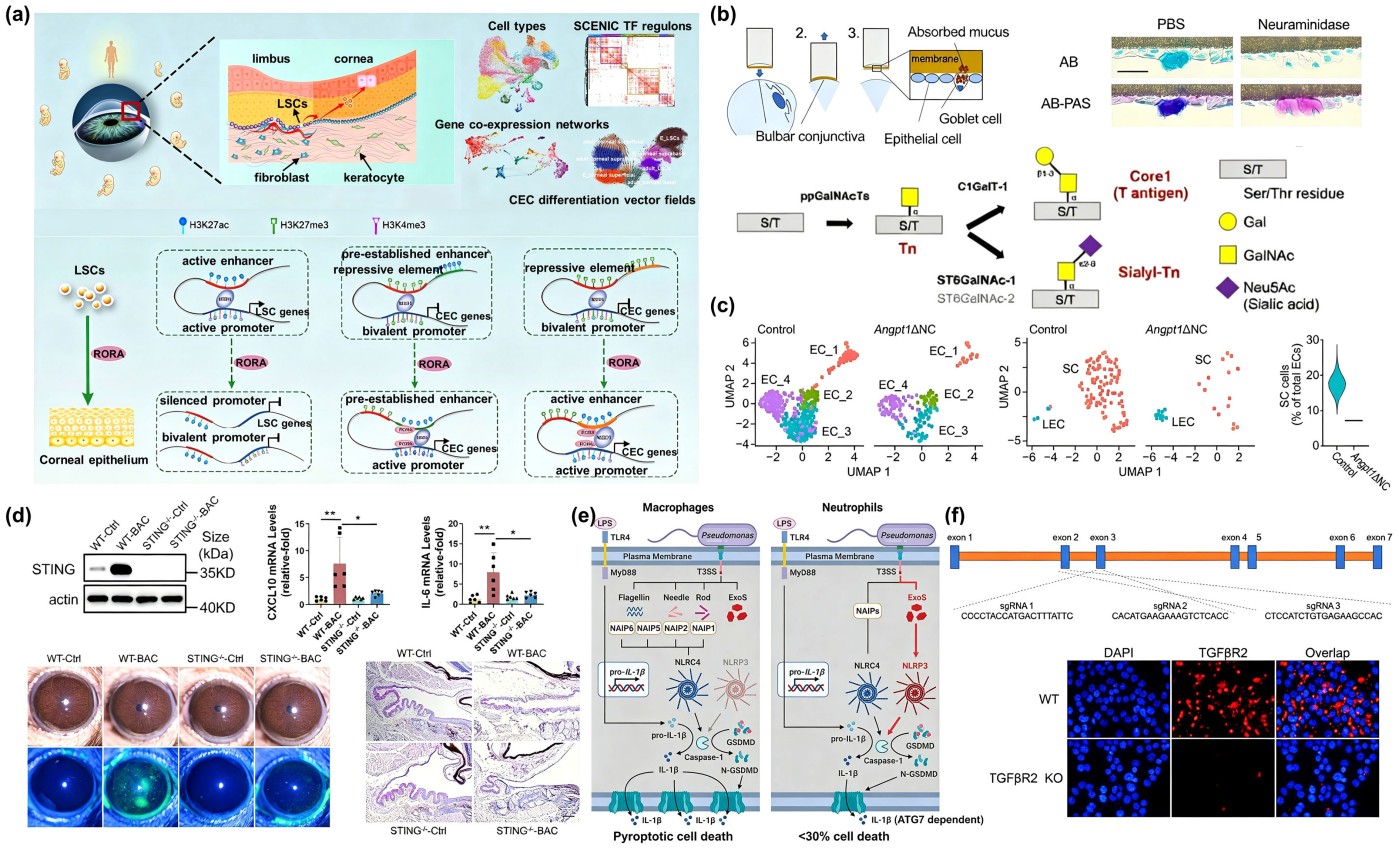
Study on representative pathology and mechanisms of AS and its associated diseases. **(A)** Regulation of LSCs differentiation and maintenance of corneal epithelial homeostasis (53). **(B)** O−glycosylation modification of conjunctival goblet cell mucins and mucosal defense barrier (54). **(C)** TM’s canal cell communication and aqueous humor dynamic homeostasis (55). **(D)** cGAS−STING pathway−mediated ocular surface inflammatory response (56). **(E)** Differential pyroptosis of immune cells regulating infectious corneal inflammation (57). **(F)** TGF−β−mediated abnormal repair of lens epithelial cells (58). *P < 0.05, **P < 0.01, ***P < 0.001.

The AS serves as a critical anatomical region that sustains light entry, refractive focusing, ocular surface homeostasis, aqueous humor circulation and IOP balance, which mainly comprises the tear film, conjunctiva, cornea, limbus, anterior chamber, iris, ciliary body, trabecular meshwork (TM), Schlemm’s canal, lens and lens capsular bag, among other structures (48–50). Rather than a single anatomical compartment, the AS constitutes a dynamic functional system jointly formed by epithelial barriers, mucus barriers, immune barriers, transparent stroma, aqueous humor kinetic pathways and postoperative tissue−repair interfaces (51, 52). By integrating single−cell transcriptomic data of human healthy corneal and conjunctival tissues, Li et al. deciphered the continuous differentiation trajectories of limbal stem cells (LSCs), basal layer, wing layer and superficial corneal epithelial cells. They also revealed that RORA−mediated three−dimensional epigenomic remodeling is involved in corneal epithelial maturation and fate maintenance, indicating that corneal transparency and epithelial barrier stability rely on elaborate hierarchical cell differentiation (53)(**Figure 2A**). From the perspective of conjunctival goblet cells, Matsuzawa et al. demonstrated that gel−forming mucins secreted by goblet cells not only participate in ocular surface lubrication but also eliminate allergens, pathogens and exogenous particles by mucin sialylation, verifying that the conjunctiva and tear film collectively form the outermost mucosal defensive barrier of the AS (54)(**Figure 2B**). Therefore, the fundamental physiological functions of the AS can be generalized into three categories, maintaining stable hydration and external defense through the tear film, conjunctiva and corneal epithelium, achieving optical transmission *via* transparent corneal stroma and regular epithelia, and sustaining aqueous humor drainage and IOP homeostasis by means of the anterior chamber, ciliary body, TM and Schlemm’s canal.

Beyond the ocular surface and cornea, the aqueous humor outflow system represents another core structure for intraocular homeostasis maintenance within the AS. Produced by the ciliary body, aqueous humor flows through the posterior chamber, pupil and anterior chamber, and is primarily drained through the conventional outflow pathway composed of the TM and Schlemm’s canal, with resistance alterations directly regulating IOP. Thomson et al. identified critical cellular crosstalk between the TM and Schlemm’s canal, wherein ANGPT1, TEK and SVEP1−related signals exert pivotal effects on Schlemm’s canal development and the integrity of the aqueous humor outflow pathway. Through genetic, histological and single−cell RNA sequencing analyses, this study validated that aberrant molecular signals derived from the TM can trigger developmental defects in Schlemm’s canal, obstruction of aqueous humor outflow and subsequent IOP elevation (55)(**Figure 2C**). This finding indicates that physiological functions of the AS rely not merely on surface homeostasis of the cornea and tear film, but also on highly ordered cellular communication and hydrodynamic balance within the iridocorneal angle region. For the therapeutic management of AS disorders, all topical pharmaceuticals, implanted biomaterials and tissue repair strategies must simultaneously take into account ocular surface clearance, corneal transparency, local immunity, aqueous humor circulation and tissue−interface responses. Otherwise, even pharmacologically active agents may fail to achieve stable therapeutic outcomes owing to poor adaptability to the complex physiological microenvironment of the AS. These anatomical features directly inform hydrogel design. Ocular-surface systems should combine adhesion with optical transparency and minimal irritation, whereas materials placed near the iridocorneal angle or lens capsule require tightly controlled swelling, mechanics, and degradation to avoid disturbing aqueous humor flow or visual quality.

### 2.2 Common pathological mechanisms of AS diseases

Although AS diseases encompass distinct pathological entities including DED, corneal injury, IK, uveitis, glaucoma and PCO, they share striking commonalities in pathological progression, namely the frequent coupling of barrier disruption, inflammatory activation, oxidative stress, immune cell recruitment, cellular apoptosis, aberrant migration, EMT and ECM remodeling. Ouyang et al. demonstrated that mitochondrial DNA can be released from the ocular surface under dryness, inflammatory stimuli and environmental stress, which further activates the cGAS−STING pathway and triggers type−I interferon−associated inflammatory responses. By linking mitochondrial damage, DNA sensing and innate immune activation, this study reveals that ocular surface disorders such as DED are not merely characterized by insufficient tear secretion, but represent inflammatory pathological processes driven jointly by epithelial stress, mitochondrial injury and abnormal mucosal immunity (56)(**Figure 2D**). Following ocular surface barrier impairment, unstable tear film, decreased mucin secretion, disrupted epithelial tight junctions and pro−inflammatory factor release can further form a vicious cycle, driving local tissues from a reversible stimulated state toward chronic inflammation and structural damage progressively.

Infection−associated AS diseases are featured by prominent interplay between pathogen invasion and host−mediated inflammatory amplification (59). Taking Pseudomonas aeruginosa keratitis as a typical example, Minns et al. verified that upon neutrophil infection by Pseudomonas aeruginosa, the pathogen selectively triggers interleukin−1β (IL−1β) release *via* the NLRP3 inflammasome, thereby modulating the severity of corneal lesions. This study further indicated that the type III secretion system (T3SS) of Pseudomonas aeruginosa and its effector proteins can regulate neutrophil responses, shifting infectious keratitis from a simple pathogen burden issue to a pathological process involving pathogen virulence, inflammasome activation and tissue injury (57)(**Figure 2E**). This evidence implies that the therapeutic management of IK should not merely focus on pathogen elimination, but also take secondary injuries into account, including neutrophil infiltration, IL−1β secretion, protease activation, stromal collagen degradation and loss of corneal transparency. For non−infectious corneal trauma, analogous inflammatory amplification and stromal remodeling can also induce keratocyte apoptosis, fibrotic transformation and myofibroblast accumulation, ultimately resulting in corneal scarring and opacification.

Postoperative AS−associated disorders and chronic aqueous humor circulation disorders are characterized by aberrant tissue repair and disrupted mechanical homeostasis. PCO serves as a typical model of abnormal AS repair following cataract surgery, in which the core pathological processes involve adhesion, proliferation, migration, EMT, and ECM deposition by residual LECs. Wang et al. demonstrated that CRISPR/Cas9−mediated knockout of TGF−β receptor II could retard the mesenchymal transition of LECs and the progression of PCO, further highlighting the pivotal role of TGF−β signaling in PCO−related EMT (58)(**Figure 2F**). Analogous to PCO, the pathological basis of glaucoma within the AS is also linked to abnormal tissue architecture and cellular behaviors, dysfunction of the TM and Schlemm’s canal elevates aqueous humor outflow resistance and consequently triggers IOP elevation. Collectively, the common mechanism underlying AS diseases is not attributed to isolated inflammation, infection, or cell proliferation. Instead, upon local barrier destabilization, inflammation, oxidative stress, altered cell fate, and ECM remodeling mutually potentiate one another, ultimately leading to compromised transparency, dysregulated aqueous humor circulation, visual axis obstruction, or long−term deterioration of visual quality. Accordingly, disease-specific hydrogel design should be matched to the dominant pathological process. Barrier-restorative and anti-inflammatory functions for DED and corneal injury, antibacterial and immunomodulatory activity for IK, and anti-adhesive, anti-migratory, and anti-EMT properties for PCO. For glaucoma-related applications, sustained drug release must be achieved without obstructing aqueous humor dynamics.

### 2.3 Common therapeutic dilemmas of AS diseases

The primary therapeutic bottleneck for AS diseases stems from rapid drug clearance and insufficient effective exposure following topical administration. Although eye drops represent the most commonly adopted administration route for the AS, instilled agents are immediately affected by tear dilution, blinking, tear turnover, and nasolacrimal drainage, resulting in limited ocular surface residence time. Garaszczuk et al. dynamically assessed tear clearance after instillation of 5 μL normal saline using anterior segment optical coherence tomography (AS−OCT), and observed a marked decline in inferior tear meniscus height at 30 s and 60 s. The average tear clearance rate reached approximately 29% and 36% respectively, which was correlated with indicators including tear film break−up time, blink frequency, and tear osmolarity (60). This clinical imaging−based evidence establishes that the local microenvironment of the AS inherently possesses strong dynamic clearance capacity. Conventional short−acting eye drops can hardly sustain continuous effective drug concentrations in target tissues associated with the cornea, conjunctiva, or anterior chamber. For hydrophilic drugs, protein−based pharmaceuticals or antibody−derived biologics, tight junctions of the corneal epithelium, conjunctival blood−mediated absorption and the BAB further restrict trans−barrier delivery, such that drug deposition on the ocular surface does not equal targeted lesion penetration.

The second therapeutic dilemma of AS diseases lies in their multi−stage pathological nature, whereby a single drug or monotherapeutic strategy fails to cover the full disease course. In IK, pathogens are capable of forming biofilms to enhance antimicrobial tolerance, followed by persistent corneal structural damage driven by inflammatory cell infiltration, protease release and stromal degradation. Using ocular−derived Staphylococcus aureus, Staphylococcus epidermidis and Candida albicans, Ranjith et al. constructed monomicrobial and polymicrobial biofilms on *in−vitro* and *ex−vivo* human corneas. Their findings validated that ocular pathogens can develop sophisticated biofilm architectures on the corneal tissue surface, and microorganisms within biofilms generally exhibit greater antimicrobial tolerance than their planktonic counterparts (61). Such outcomes reveal that IK treatment confronts not only challenges of drug penetration and pathogen eradication, but also sequential pathological processes including biofilm barrier formation, inflammatory exacerbation, stromal injury and scar−associated repair. Similarly, corneal trauma, PCO and uveitis are not lesions occurring at a single time point. Instead, they undergo a dynamic progression encompassing early injury stimulation, intermediate inflammatory amplification, and late−phase cell migration and tissue remodeling. Accordingly, merely elevating drug concentrations may not simultaneously satisfy the demands of anti−inflammation, anti−infection, pro−regeneration and anti−fibrosis.

Long−term management requirements and insufficient patient compliance constitute the third therapeutic dilemma of chronic AS diseases. Disorders such as DED, glaucoma, and recurrent uveitis usually require long−term regular medication, whereas frequent eye drop instillation tends to cause missed doses, treatment discontinuation, dosage fluctuations, and unstable therapeutic outcomes. Medeiros et al. conducted the phase−III randomized ARTEMIS 1 trial to compare the efficacy of degradable bimatoprost implants versus twice−daily timolol eye drops in patients with open−angle glaucoma and ocular hypertension. Designed as a 20-month, multicenter, randomized, masked, parallel−controlled study with repeated dosing cycles, this trial aimed to verify whether long−acting implantable systems could replace part of high−frequency topical drop therapies (62). Such clinical trials reflect a core challenge in the management of chronic AS disorders, effective pharmaceuticals do not necessarily guarantee long−term therapeutic benefits, and treatment outcomes are equally determined by administration frequency, local tolerability, sustained−release capacity, and patient adherence. Overall, therapeutic bottlenecks of AS diseases mainly converge on three dimensions, inadequate drug exposure resulting from rapid local clearance and barrier constraints, the incapacity of single interventions to cover multi−stage pathological courses, and decreased compliance induced by the burden of long−term medication. Therefore, subsequent therapeutic strategies should shift from simple “drug administration” to an integrated intervention mode that places equal emphasis on stable retention, effective penetration, disease−course matching, and long−term management. These therapeutic constraints favor hydrogels that integrate retention, barrier-compatible penetration, and stage-matched release rather than simply increasing the administered dose. They also indicate that the dosage form should be selected according to the clinical scenario, including *in situ* gels for prolonged topical exposure, transparent scaffolds for corneal repair, contact-lens-based depots for chronic therapy, and intracapsular systems for postoperative prophylaxis.

## 3 Hydrogel-based loading, release, and repair as three core functional modules for AS diseases

“Loading” refers to the incorporation and stabilization of therapeutic or functional agents within a hydrogel. “Release” describes control over their rate, duration, sequence, and stimulus responsiveness, whereas “repair” denotes restoration of tissue structure and function through released agents, intrinsic material activity, or both. These modules are interconnected but not strictly dependent, as hydrogels may also promote repair through adhesion, ECM mimicry, cytokine sequestration, and microenvironment regulation. Their relative importance varies by disease. DED emphasizes lubrication, retention, and anti-inflammatory or antioxidant effects. Corneal injury requires transparency, low swelling, wet adhesion, and ECM-like support. IK requires rapid antimicrobial activity with immunomodulatory and regenerative effects. Glaucoma prioritizes sustained release with minimal visual and mechanical interference, whereas PCO prevention focuses on inhibiting adhesion, migration, and EMT within the capsular bag.

The core value of hydrogels in the treatment of AS diseases lies not merely in prolonging drug retention time, but more importantly in their capability to systematically achieve therapeutic factor loading, release regulation, and tissue microenvironment remodeling by virtue of three−dimensional hydrophilic networks, tunable porous architectures, wet−tissue adhesive interfaces, dynamic cross−linking bonds, stimuli−responsive features, and ECM−mimicking structures. Compared with conventional eye drops, eye ointments, or passive postoperative interventions, hydrogels are capable of constructing a stable local therapeutic platform under multiple constraints, including tear dilution, blink−mediated clearance, nasolacrimal drainage, corneal epithelial tight junctions, BAB restriction, and postoperative inflammatory microenvironments. Its functional cascade can be summarized as three sequential processes, loading, release, and repair. First, hydrogels accommodate small−molecule drugs, biomacromolecules, nanozymes, antioxidant nanoparticles, or functionalized interfaces through their highly hydrated, porous, and functionalizable networks. Second, hydrogels regulate spatiotemporal release of therapeutic agents through diffusion restriction, dynamic covalent interactions, affinity complexation, micro−nano composite structures, and environment−responsive mechanisms. Finally, hydrogels participate in tissue reconstruction and pathological microenvironment remodeling through wet−tissue adhesion, ECM biomimicry, modulation of cellular behavior, cytokine sequestration, antioxidant, anti-inflammatory, anti-infective, and anti-fibrotic effects. Hence, hydrogels applied in AS disease treatment should be regarded as a material−network−driven proactive therapeutic platform rather than a simple drug delivery system. The representative hydrogel systems have also been summarized (**Table 1**).

**Table 1:** Representative hydrogel systems for AS disease therapy.

Disease	Hydrogel system	Core design	Main function	Major advantage	Ref
DED	AF127/Cu_2-X_Se	Adhesive thermogel carrying Cu_2-X_Se nanozymes	ROS scavenging, anti-inflammatory activity, tear-film protection	Prolonged ocular retention	(63)
Uveitis	Chitosan/β-GP/ADA	Mucoadhesive *in situ* gel for adalimumab	TNF-α inhibition	Non-invasive biologic delivery	(64)
Uveitis	F127/Ce-MOFs/DSP	Thermogel with DSP-loaded Ce-MOFs	Anti-inflammation, antioxidation	Combined drug delivery and ROS control	(65)
Corneal injury	Hep@Gel	EBM-mimetic GelMA-heparin network	Cytokine sequestration, anti-fibrosis	Drug-free scar prevention	(66)
Corneal defect	pDCSM/HAMA	Transparent ECM-mimetic scaffold	Re-epithelialization, regeneration	Low-swelling, sutureless repair	(67)
IK	SQPV	Dual network with PDRN and verteporfin micelles	Antibacterial, regenerative, anti-fibrotic	Stage-matched sequential release	(68)
IK	SDF-1/QUDCS/OD	Self-healing hydrogel with an SDF-1 gradient	Antimicrobial action, stem-cell recruitment	Infection control with endogenous repair	(69)
Glaucoma	Gellan gum/chitosan/timolol	Mucoadhesive *in situ* gel depot	Sustained IOP lowering	Longer residence, reduced dosing	(70)
PCO	HCM_6_/Met	Injectable pH-responsive dynamic hydrogel	Inhibits LEC proliferation, migration, and EMT	pH-adaptive intracapsular release	(71)
PCO	Zwitterionic cellulose IOL	Foldable antifouling hydrogel IOL	Suppresses LEC adhesion and migration	Drug-free prevention with optical clarity	(72)

DED, dry eye disease; IK, infectious keratitis; IOL, intraocular lens; LEC, lens epithelial cell; PCO, posterior capsule opacification; ROS, reactive oxygen species.

### 3.1 “Loading” based on hydrogel systems

**Figure 3:**
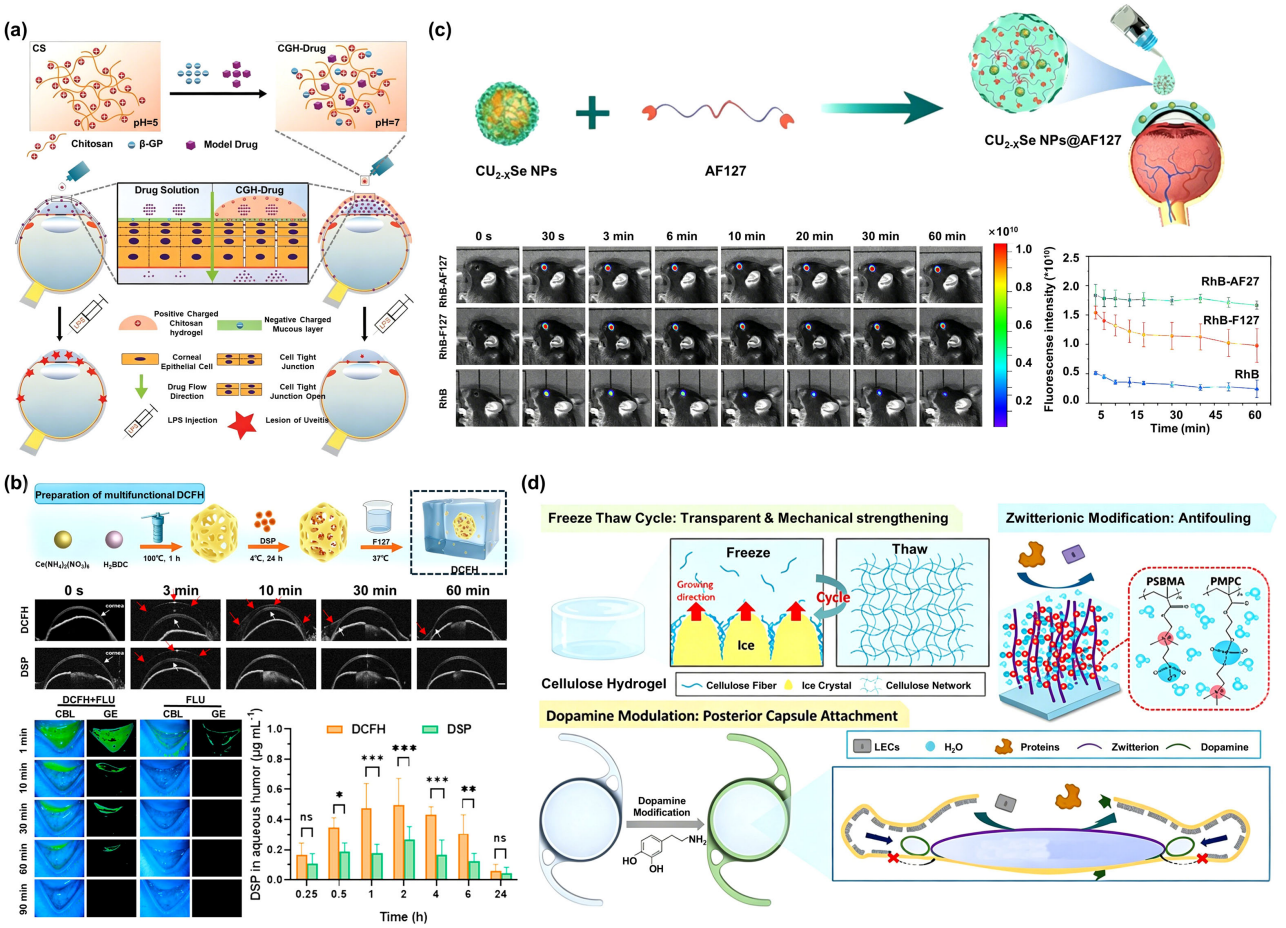
Hydrogel−based loading strategies for AS therapy. **(A)** Schematic of chitosan/β−glycerophosphate hydrogel−mediated adalimumab delivery for enhanced ocular retention and permeability (64). **(B)** Preparation and uveitis−targeting performance of multifunctional DCFH hydrogel loaded with DSP and Ce−MOFs (65). **(C)** AF127 hydrogel−anchored Cu_2-X_Se nanozymes for long−term ocular antioxidative therapy *via* Schiff−base−mediated adhesion (63). **(D)** Design of zwitterionic−modified cellulose hydrogel IOL for inhibiting lens epithelial cell migration and preventing PCO (72). NS: not significant, P > 0.05. *P < 0.05, **P < 0.01, ***P < 0.001.

The “loading” function of hydrogels is first reflected in their ability to form a local reservoir of therapeutic factors through three-dimensional hydrophilic networks and tunable porous structures (73, 74). AS tissues are chronically in a dynamic liquid environment, conventional eye drops are prone to being affected by tear turnover, blinking, and nasolacrimal drainage after administration, leading to short local retention time and low effective drug concentration. In contrast, hydrogels can utilize their high water content, porous architectures, and flexible networks to encapsulate, adsorb, or stably embed therapeutic factors within the material, transforming drugs from one-time rapid exposure into a local reservoir-based state. This loading capacity is particularly crucial for hydrophilic drugs, biomacromolecules, and protein-based pharmaceuticals (73–77). For example, chitosan/β-glycerophosphate hydrogel eye drops are used to load adalimumab (ADA). This system leverages the biocompatibility, hydrophilicity, mucosal adhesiveness, and easy-loading properties of chitosan, converting ADA from traditional systemic administration or intraocular injection into a local non-invasive delivery format. Studies have implied that chitosan-based hydrogels can interact with the tear film mucin layer using their positively charged amino groups, thereby enhancing ocular surface retention and improving drug penetration. When ADA is encapsulated in hydrogels, it exhibits better penetration efficiency and therapeutic efficacy compared with free drugs (64)(**Figure 3A**). This work exhibits that the “loading” of hydrogels is not a simple drug mixing process but rather changes the local existence mode of macromolecular drugs in the AS through the material network, enabling the local application of biologics that were originally difficult to deliver effectively by way of eye drops.

Hydrogels can also construct multi−functional loading platforms upon integration with nanomaterials. Functional nanoparticles, metal−organic frameworks (MOFs), and nanozymes frequently possess antioxidant, anti−inflammatory, antibacterial, or drug−adsorptive capacities, yet their standalone ocular application is restricted by rapid clearance, particle aggregation, or local irritation. After incorporation into hydrogel networks, hydrogels offer physical immobilization and local retention, while acting as secondary carriers for nano−components to boost their stability and safety for ophthalmic use (78, 79). Taking DCFH multi−functional F127 hydrogel eye drops as an example, this system loads dexamethasone sodium phosphate (DSP) into ROS−scavenging Ce−MOFs, followed by embedding the composites into thermosensitive F127 hydrogels. Ce−MOFs serve as both functional drug carriers and antioxidant moieties, whereas F127 elevates ocular surface retention and anterior chamber bioavailability of therapeutics on account of its body−temperature−triggered *in−situ* gelation, thixotropy and optical transparency (65)(**Figure 3B**). This system represents an advanced form of hydrogel−mediated “loading”, namely the integration of small−molecule drugs, functional nanocarriers and thermosensitive polymer networks into synergistic therapeutic platforms.

Beyond physical encapsulation and nanocomposite construction, hydrogels can also achieve localized anchoring of therapeutic factors through functional group modification. Given the humid and highly fluid nature of the ocular surface environment, merely increasing viscosity is often insufficient to realize long−term retention. Modified with functional moieties such as aldehyde, catechol, amino, carboxyl, heparin, or zwitterionic groups, hydrogels can establish reversible or stable interfacial interactions with ocular surface mucins, proteins, and the ECM, thereby improving spatial localization (80, 81). This strategy is exemplified by a study on aldehyde−functionalized F127 (AF127) hydrogels for delivering Cu_2-X_Se nanoparticles. F127 features favorable biosafety, stability, injectability, and thermosensitivity. Its terminal hydroxyl groups can be functionalized into aldehyde groups. Since ocular surface tear fluid is rich in mucins, lipids, peptides, and proteins, aldehyde groups can form Schiff base linkages with amino groups within protein molecules, enhancing ocular surface adhesion and retention. In this research, Cu_2-X_Se nanoparticles with superoxide dismutase (SOD)− and glutathione peroxidase (GPX)−mimicking catalytic activities were loaded into AF127 hydrogels, constructing a localized therapeutic platform capable of both nanozyme loading and tissue adhesion (63)(**Figure 3C**). This design illustrates that the “loading” function of hydrogels involves not only the encapsulation of therapeutic agents but also their retention at targeted anatomical sites.

The “loading” function of hydrogels can also be extended from drug delivery to the fabrication of functionalized material interfaces. For AS diseases, therapeutic platforms are not restricted to eye drops or *in−situ* hydrogels. They can also be contact−based devices, intracapsular drug reservoirs, corneal substitute scaffolds, or functionalized IOLs (82, 83). Foldable cellulose−based hydrogel IOLs modified with zwitterions and reinforced through freeze−thaw cycles demonstrate the loading capacity of hydrogels as functional implantable interfaces. Mechanical strength and optical transparency of the material are elevated through repeated freeze−thaw treatment. Zwitterionic modification endows the surface with anti−fouling and anti−cell−adhesive properties, while dopamine is grafted onto the haptics and peripheral posterior surface to strengthen adhesion to the posterior capsule. Such design blocks the migration of LECs without compromising central optical transparency (72)(**Figure 3D**). Consequently, hydrogel−mediated loading in AS diseases should be interpreted as a continuous process ranging from molecular immobilization and nanocomponent incorporation to macroscale functional interface construction. Essentially, therapeutic functions are stably delivered into localized lesion microenvironments through the rational design of material networks and interfacial properties.

### 3.2 “Release” based on hydrogel systems

While the “loading” strategy addresses how therapeutic agents are encapsulated and stably localized within hydrogels, the “release” profile governs the rate, sequence, and environment-responsive modes by which these agents exert their therapeutic effects (70, 84–88). AS diseases are frequently characterized by chronicity, recurrence, or progressive postoperative pathological changes. Short-term high-dose exposure fails to cover the entire pathological course, whereas burst release may induce localized toxicity or off-target irritation (84, 85, 87). Therefore, the “release” function of hydrogels is not merely passive retardation of drug diffusion. Instead, it achieves reprogramming of spatiotemporal behaviors of therapeutic factors with well−designed network architecture, intermolecular interactions, and stimulus−responsive mechanisms. Basal release kinetics are primarily regulated by hydrogel pore size, crosslinking density, swelling behavior, and degradation rate. Advanced release systems can match release patterns to ocular pathological microenvironments and tissue repair stages using dynamic covalent bonds, affinity complexation, micro−/nanocomposite drug depots, pH−responsiveness, photoresponsiveness, or sequential release architectures.

**Figure 4:**
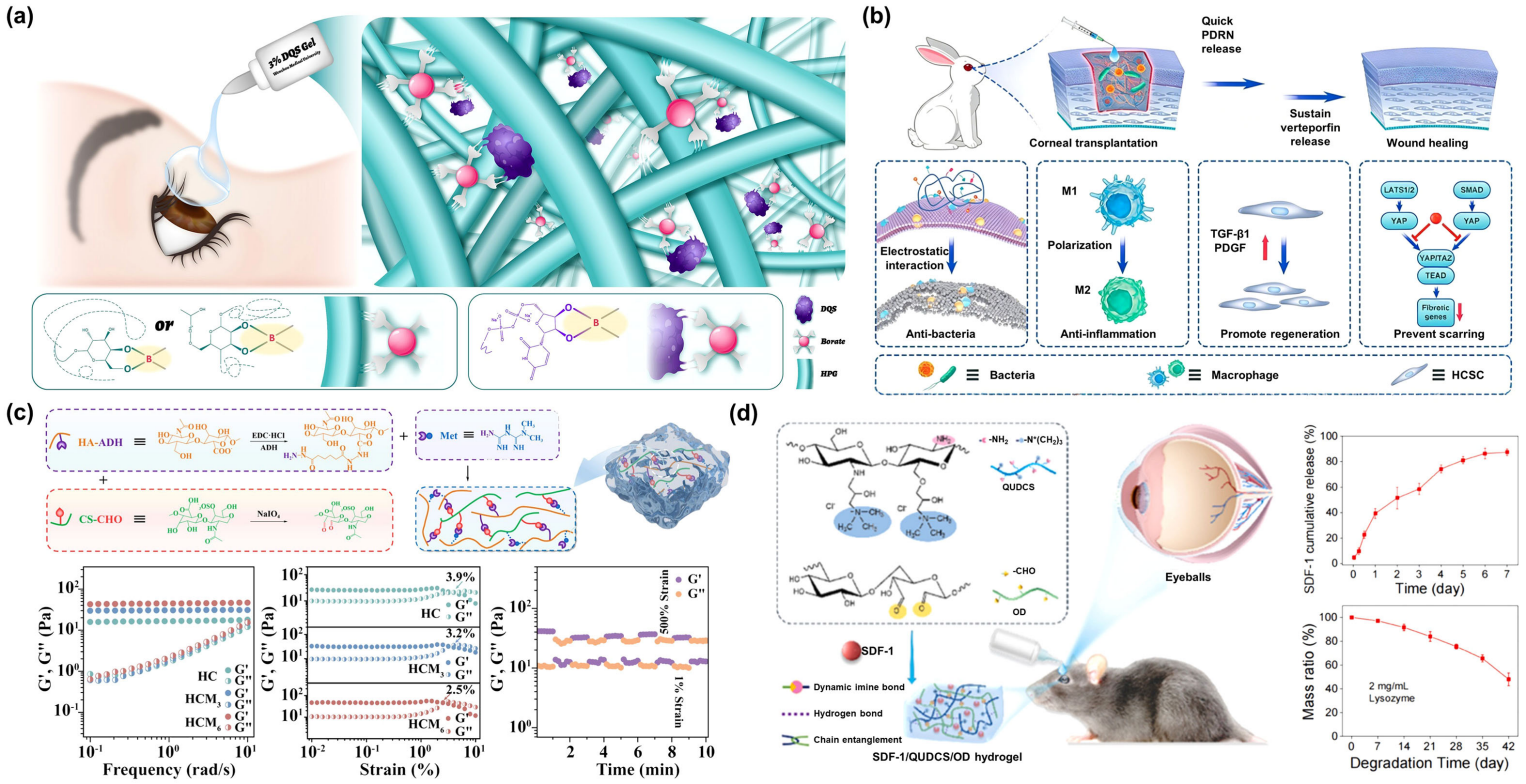
Hydrogel−mediated controlled release systems for AS therapy. **(A)** Dynamic covalent complexation−based DQS hydrogel for sustained ocular delivery of diquafosol tetrasodium (89). **(B)** SQPV dual−network hydrogel for sequential and spatiotemporal release of PDRN and verteporfin (68). **(C)** pH−responsive HCM_6_ hydrogel for on−demand metformin release tailored to pathological microenvironmental acidosis (69). **(D)** SDF−1/QUDCS/OD hydrogel for sustained chemokine delivery and chemotactic gradient formation to recruit endogenous stem cells (71).

Dynamic covalent bonds or affinity interactions serve as vital strategies for hydrogels to modulate release profiles, particularly for hydrophilic small−molecule drugs. Hydrophilic agents generally dissolve readily in tear fluid or aqueous humor, making long−term sustained release hard to achieve through hydrophobic interactions or crystalline dissolution. DQS−affinity hydrogels offer a representative solution to this challenge. As a hydrophilic small molecule bearing a cis−diol moiety, DQS forms dynamic covalent complexes with borate groups. Researchers constructed a 3% DQS Gel through borate−mediated dynamic covalent complexation between DQS and hydroxypropyl guar (HPG). Within this system, HPG−borate interactions act as crosslinking junctions to form the hydrogel network, whereas DQS−borate interactions function as anchoring sites to temporarily confine DQS inside the network. Compared with the free DQS solution, the 3% DQS Gel exhibits slowed initial release, sustained late−stage drug concentration, reduced corneal penetration, and enhanced ocular surface enrichment. Given the low molecular weight of DQS, conventional pore−trapping mechanisms cannot fully account for its sustained−release performance, the prolonged release mainly originates from dynamic covalent complexation between DQS and the hydrogel network (89)(**Figure 4A**). This work demonstrates that hydrogels can transform release behaviors from simple physical diffusion to molecular recognition−regulated release through the dynamic processes of binding, dissociation, and diffusion.

Micro−nanocomposite architectures facilitate the formation of hierarchical drug depots within hydrogels, extending release pathways and regulating release sequences. The SQPV hydrogel serves as a typical representative. This system constructs a dual−network structure with methacrylated silk fibroin (SFMA) and quaternized chitosan derivatives, while integrating verteporfin−loaded PLGA−PEG micelles and introducing polydeoxyribonucleotide (PDRN). Its core design disperses hydrophilic PDRN within the hydrogel skeleton and encapsulates hydrophobic verteporfin inside micelles, such that the two types of drugs are controlled by separate release routes. Experimental results demonstrate that PDRN is almost completely released within 7 days, while only around 72% of verteporfin is liberated over 28 days, showing obvious two−stage and sequential release profiles. Such spatiotemporally controllable release first relieves inflammation and accelerates tissue regeneration with PDRN, and subsequently modulates fibrotic remodeling using verteporfin, matching different phases of tissue repair (68)(**Figure 4B**). This system indicates that release behaviors of hydrogels can advance from single sustained release to multi−component, multi−pathway, and staged release, establishing a correlation between material release kinetics and tissue healing processes.

Stimulus−responsive release enables hydrogels to sense pathological microenvironments and deliver drugs on demand. Following AS surgery or under inflammatory conditions, local pH values, ROS levels, enzymatic activities, and inflammatory factors may alter, and such variations act as endogenous signals to trigger drug release. The pH−responsive metformin (Met)−loaded hydrogel HCM_6_ exemplifies this strategy. This hydrogel is fabricated from dynamic covalent crosslinking among modified hyaluronic acid−adipic dihydrazide (HA−ADH), oxidized chondroitin sulfate (CS−CHO) and Met, eliminating toxic crosslinkers and tuning Met release alongside local pH shifts. Studies have indicated that the pH of normal aqueous humor generally remains between 7.32 and 7.60, whereas microenvironments associated with postoperative conditions or abnormal lens epithelial cell (LEC) proliferation tend to be acidified. Thus, HCM_6_ is engineered as an injectable hydrogel adaptive to microenvironmental pH fluctuations. Rheological characterizations confirm its stable gel state, shear−thinning behavior, self−healing capacity and injectability, revealing that the dynamic network facilitates practical administration and provides structural foundations for responsive release (71)(**Figure 4C**). This design couples release profiles with lesion microenvironments, shifting hydrogel release modes from preprogrammed patterns toward environment−responsive delivery. However, stimulus responsiveness demonstrated in buffered solutions does not necessarily translate directly *in vivo*. The magnitude and duration of acidosis, oxidative stress, enzyme activity, and inflammatory signaling vary with disease stage, microbial burden, concomitant therapy, and patient-specific tear or aqueous humor composition. Consequently, responsive thresholds established *in vitro* may produce incomplete activation, premature release, or substantial inter-individual variability *in vivo*. Future studies should therefore quantify disease-relevant trigger ranges and validate responsiveness in dynamic ocular models.

For protein factors or chemokines, hydrogel−mediated release focuses not only on prolonged therapeutic duration but also on generating spatial gradients at target sites. The SDF−1/QUDCS/OD hydrogel forms an injectable and self−healing network upon Schiff−base dynamic crosslinking between quaternized ultra−highly deacetylated chitosan (QUDCS) and oxidized dextran, with stromal cell−derived factor−1 (SDF−1) encapsulated within the matrix. Elevated QUDCS concentrations reduce hydrogel pore size while boosting crosslinking density and mechanical strength, enabling protein encapsulation without compromising the permeability required for corneal metabolism. Researchers further optimized SDF−1 loading concentrations to sustain effective dosages over a 7−day therapeutic cycle, with continuous release generating chemotactic gradients. Such release patterns not only maintain therapeutic factor concentrations but also establish spatial signals regulated by the SDF−1/CXCR4 axis through local retention, sustained liberation and bioactivity preservation, accelerating cell recruitment and tissue repair (69)(**Figure 4D**). Overall, hydrogels advance therapeutic factor release from passive diffusion toward spatiotemporally programmable delivery by adopting strategies including dynamic complexation, micro−nanostructural integration, pH responsiveness and gradient release. This constitutes a core advantage that distinguishes hydrogels from conventional eye drops and single−carrier systems.

Common *in vitro* release tests provide only an approximation of ocular performance. Static PBS-based dialysis or immersion assays do not reproduce tear turnover, blinking-induced shear, protein and mucin adsorption, corneal uptake, enzymatic degradation, inflammatory mediators, or the small and dynamically renewed fluid volumes of the ocular surface and anterior chamber. Release data should therefore be interpreted together with ex vivo permeation, dynamic flow models, ocular pharmacokinetics, retention imaging, and disease-specific *in vivo* efficacy rather than as a stand-alone predictor of clinical exposure. The design priorities of sustained release also differ among chronic uveitis, glaucoma, and PCO. In uveitis, thermosensitive or microenvironment-responsive depots are intended to prolong local corticosteroid or biologic exposure while limiting repeated intraocular administration. In glaucoma, *in situ* gelling formulations and contact-lens-based reservoirs seek to maintain IOP-lowering drug concentrations over extended periods and reduce dosing frequency. In PCO prevention, intracapsular hydrogels and functionalized IOL interfaces must confine release or anti-cellular activity to the capsular bag, because excessive diffusion or swelling may compromise surrounding ocular tissues and optical performance.

### 3.3 “Repair” based on hydrogel systems

The repair performance of hydrogels represents the segment that best reflects the proactive therapeutic features of materials within the “loading−release−repair” functional cascade. For AS diseases, therapeutic endpoints are not merely drug accumulation at lesion sites or reduced inflammatory markers. They involve the restoration of corneal transparency, reconstruction of epithelial barriers, ordering of stromal architecture, suppression of pathological fibrosis, infection control, relief of oxidative stress, and recovery of local homeostasis (90, 91). Consequently, the repair capacity of hydrogels should not be narrowly interpreted as wound coverage. Instead, it refers to the process whereby materials modulate tissue microenvironments on the basis of their intrinsic structure, interfacial properties, and bioactivities. Compared with ordinary dressings or inert filling materials, repair−oriented hydrogels not only supply moist tissue adhesion and structural support but also actively interfere with pathological wound−healing cascades through ECM biomimicry, cytokine sequestration, antioxidation, and anti−infection mechanisms (90, 92, 93). Two mechanisms should be distinguished. Drug-mediated repair is produced primarily by released antibiotics, anti-inflammatory agents, growth factors, or anti-fibrotic compounds, whereas material-intrinsic repair arises from the hydrogel itself through wet adhesion, ECM mimicry, cytokine sequestration, antioxidative activity, barrier reconstruction, or regulation of cell behavior, even in the absence of conventional drug loading. Many advanced systems combine both mechanisms and should be evaluated accordingly.

**Figure 5:**
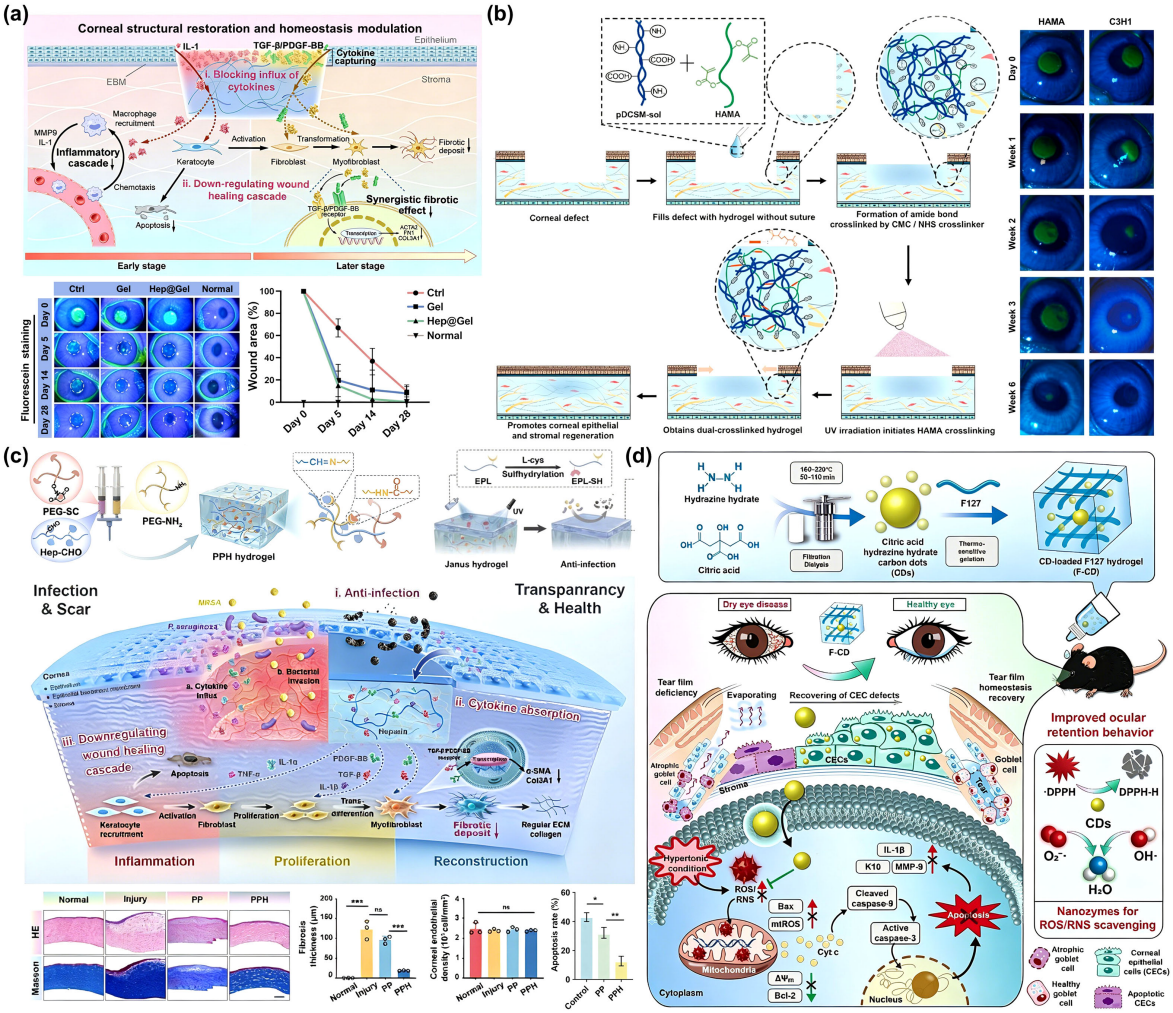
Hydrogel-mediated repair strategies for AS diseases. **(A)** Hep@Gel hydrogel mimics epithelial basement membrane to sequester pro-fibrotic cytokines and prevent corneal scarring (66). **(B)** pDCSM/HAMA dual-crosslinked hydrogel as ECM-mimetic scaffold for corneal defect repair and regeneration (67). **(C)** EPPH Janus hydrogel with bilayered design for synergistic anti-fibrotic regulation and antibacterial protection (94). **(D)** F-CD thermosensitive hydrogel for ocular surface microenvironment remodeling *via* antioxidative and anti-inflammatory effects (95).

Basement membrane biomimicry and cytokine sequestration serve as key mechanisms underlying scar−free repair mediated by hydrogels. Under physiological conditions, the corneal epithelial basement membrane (EBM) functions not only as a structural barrier but also as a critical interface restricting the diffusion of epithelium−derived inflammatory and fibrotic factors toward the stromal layer. Once the EBM gets damaged, factors including IL−1, TGF−β and PDGF−BB penetrate the stroma, triggering keratocyte apoptosis, fibrotic transformation and myofibroblastic transdifferentiation, which ultimately lead to scar formation and impaired corneal transparency. Inspired by the EBM structure, the bioactive Hep@Gel hydrogel is fabricated from gelatin methacryloyl (GelMA) and highly anionic heparin, mimicking collagen networks and heparan sulfate proteoglycan−like structures within native EBM. Experimental data demonstrate that Hep@Gel is capable of sequestering IL−1, TGF−β, and PDGF−BB. Specifically, Hep10@Gel binds 65.3% of IL−1, 45.7% of TGF−β, and 57.6% of PDGF−BB within 24 h, recovering EBM−mimetic barrier performance by confining the spread of these factors. In an *in−vitro* corneal injury model, Hep@Gel blocks 83.8% of IL−1 from infiltrating the lower compartment and markedly alleviates keratocyte apoptosis. Meanwhile, its obstruction against TGF−β and PDGF−BB suppresses the transdifferentiation of keratocytes into myofibroblasts (66)(**Figure 5A**). These findings reveal that hydrogel−driven repair can be achieved independent of drug release. Instead, such materials mitigate scar formation at the source by sequestering detrimental cytokines, reconstructing biological barriers and interrupting abnormal repair cascades.

ECM−biomimetic scaffolds represent another vital route for hydrogels to participate in tissue repair. Repair of transparent AS tissues demands materials that not only fill tissue defects but also possess transparency, mechanical strength, low swelling ratio, slow degradation rate, and cell−supporting capacity comparable to native tissues. The dual−crosslinked pDCSM/HAMA hydrogel integrates porcine decellularized corneal stroma matrix (pDCSM) with methacrylated hyaluronic acid (HAMA), achieving integration of bioactivity and structural stability. Herein, pDCSM delivers cell−adhesive and regenerative signals derived from corneal ECM, whereas HAMA sustains hydrogel shape and mechanical robustness. Pre−gel solutions can directly fill irregular corneal defects and tightly adhere to stromal beds after short−term photocrosslinking. This dual−network structure features high visible−light transmittance, low swelling and slow degradation, enabling long−term retention at corneal defect sites and facilitation of tissue regeneration. Stable amide bonds form not only between amino and carboxyl groups inside pDCSM but also through reactions with adjacent tissues, raising adhesive strength between hydrogels and host tissues. Relative to pure HAMA hydrogels, the pDCSM component markedly improves cell adhesion and re−epithelialization capacity. The dual−crosslinked hydrogel supports keratocyte adhesion, survival and proliferation (67)(**Figure 5B**). This study indicates that repair−oriented hydrogels should satisfy both structural substitution and biological induction requirements. In other words, ECM−biomimetic networks furnish suitable microenvironments for cell migration, phenotype maintenance, and tissue integration.

Spatially stratified architectures further expand the repair capacity of hydrogels. Conventional homogeneous hydrogels rarely satisfy anti−fibrotic demands for inner tissue regions and anti−infection requirements for outer surfaces simultaneously. Janus hydrogels realize differentiated functions at distinct interfaces within a single material by assigning separate roles to upper and lower layers. The EPPH Janus hydrogel consists of a bottom PPH layer and a top EPL layer. The bottom PPH layer forms upon *in−situ* crosslinking between four−arm PEG and negatively charged heparin. It mainly downregulates wound−healing cascades, suppresses corneal fibrosis and restores transparency following electrostatic adsorption of cytokines secreted by injured epithelia with heparin. The top EPL layer acts as an antibacterial barrier against infections caused by methicillin−resistant Staphylococcus aureus (MRSA) and Pseudomonas aeruginosa. This research demonstrates that PPH hydrogels possess favorable optical transparency, mechanical strength, bioadhesion and biocompatibility. Negatively charged heparin within the material sequesters cytokines released from damaged corneal epithelia to restrain keratocyte apoptosis and transdifferentiation. The upper EPL layer delivers prominent antibacterial performance and reduces fibrosis by 84.8% in corneal defect models (94)(**Figure 5C**). Such stratified structures verify that hydrogel−mediated repair can be achieved through spatial programming. The matrix−adjacent side modulates fibrotic microenvironments, while the externally exposed side provides antibacterial protection.

Distinct from structural and fibrotic regulation, immune−targeted microenvironment reprogramming has emerged as a core repair strategy for infectious AS disorders, especially bacterial keratitis driven by dysregulated innate immunity. A representative ROS−responsive hydrogel eye drop system (TPG@OC) has been established to synchronize deep−tissue antibacterial efficacy and targeted immunomodulation *via* the HMGB1-neutrophil-NETs inflammatory amplification axis. This platform integrates glycyrrhizic acid−mediated HMGB1 neutralization, ROS scavenging capability, and cornea−penetrating micelles for transcytosis−dependent antibiotic delivery into the deep stroma. In response to the oxidative and inflammatory pathological microenvironment, the hydrogel undergoes intelligent on−demand degradation to release therapeutic agents, inhibit TLR4/NF−κB signaling activation, mitigate neutrophil infiltration, and restrain excessive neutrophil extracellular traps (NETs) formation. By interrupting the self−reinforcing inflammatory feedback loop, this design effectively alleviates stromal destruction, preserves corneal biomechanical stability, and maintains tissue transparency (96). Such immune−regulatory hydrogels extend repair functions beyond passive physical support and anti−infective effects, enabling active reshaping of the ocular immune microenvironment, which represents a pivotal advancement for immunology−driven therapeutic development in ocular infectious diseases.

Beyond structural repair and fibrosis modulation, microenvironmental remodeling featuring antioxidant, anti-inflammatory, and anti-apoptotic effects represents another key direction for hydrogels to boost functional recovery of AS tissues. Excessive ROS accumulation, upregulated inflammatory factors, disrupted epithelial barriers, and elevated cell apoptosis are common pathological processes in numerous AS disorders. The thermosensitive antioxidant carbon−dot−loaded F−CD hydrogel is engineered targeting such shared microenvironmental features. This system incorporates metal−free carbon nanodots (CDs) into F127−based thermosensitive hydrogels. *In−situ* gelation of F127 on the ocular surface prolongs CD retention, whereas CDs scavenge free radicals, including OHO_2_^¯^ and DPPH, to exert antioxidative, anti−inflammatory, and anti−apoptotic effects. Experimental findings reveal that the F−CD hydrogel accelerates epithelial repair, increases corneal epithelial thickness, stimulates tear secretion, and elevates goblet cell counts. Further mechanistic analyses indicate that the F−CD hydrogel markedly reduces IL−1β levels, suppresses K10, a marker of corneal epithelial squamous metaplasia, and achieves approximately 97% inhibition against MMP−9 linked to corneal barrier dysfunction, surface irregularity, and cell apoptosis. Hence, the repair performance of this system does not rely merely on drug release. Thermosensitive hydrogels extend the exposure duration of functional nanomaterials, which remodel the ocular surface microenvironment from multiple dimensions, including ROS scavenging, inflammation relief, apoptosis suppression, and epithelial barrier reconstruction (95)(**Figure 5D**). Compared with “loading” and “release”, “repair” places greater emphasis on active regulation of tissue fate and local microenvironments by the material itself. Future hydrogel designs for AS disease treatment shall evolve from simple drug loading or sustained−release strategies toward integrated platforms combining material architecture, release profiles, and tissue repair. Such hydrogels can not only deliver therapeutic agents to lesion sites but also participate in tissue reconstruction, barrier recovery, and long−term homeostasis maintenance in response to pathological microenvironmental demands.

## 4 Conclusions and future perspectives

### 4.1 Conclusions

Hydrogels have surpassed conventional sustained−release drug carriers in AS disease therapy, evolving into multifunctional therapeutic platforms integrating local delivery, intelligent release control, tissue adaptation and microenvironment remodeling (3, 6, 10). Their high water content, flexible mechanical properties, favorable transparency, tunable degradation, tissue adhesion and stimulus−responsive characteristics enable superior adaptation to the humid, soft and transparent special microenvironment of the ocular surface and intraocular regions (6, 10). Centered on the three−module functional chain of “loading-release-repair”, hydrogels support customized design tailored to the pathological features of distinct AS disorders. For DED, hydrogels extend ocular surface retention, improve lubrication, restore mucin homeostasis, and mitigate oxidative stress (63). For corneal injuries, hydrogels deliver transparent wet adhesion, ECM−mimetic support, barrier reconstruction, and anti−scar repair capacity. For IK, hydrogels integrate antibacterial, anti−inflammatory, immunoregulatory, pro−regenerative, and anti−fibrotic functions, aligning therapeutic procedures with continuous repair demands of infected wounds from pathogen clearance to tissue reconstruction (22). For uveitis, hydrogels enhance local drug retention and trans−barrier delivery efficiency, laying material foundations for non−invasive anti−inflammatory treatment and synergistic anti−inflammatory−antioxidant intervention. For glaucoma, drug−loaded hydrogel devices prolong IOP−lowering drug release duration, reduce administration frequency and improve compliance for long−term management (44). For PCO prevention, hydrogels actively regulate adhesion, migration, proliferation and EMT of residual LECs through intracapsular local delivery, antifouling surfaces, spatial isolation and functionalized IOLs design (31). Generally, the core value of hydrogels lies in the integration of drug loading, release regulation, tissue repair, immune microenvironment intervention, and pathological microenvironment intervention within a single platform. Such innovation transforms AS disease therapy from short−acting, monotypic, and passive local administration modes toward long−acting, precise, and proactive comprehensive therapeutic strategies.

### 4.2 Challenges and future perspectives

Despite promising prospects of ocular hydrogels in AS disease treatment, their clinical translation confronts multiple challenges regarding material performance, biosafety, and disease adaptability (3, 6, 10). For applications such as corneal repair and hydrogel−based IOLs, materials must strike a balance among high transparency, low haze, appropriate mechanical strength, low swelling ratio, and controllable degradation. Natural polymer hydrogels generally exhibit favorable biocompatibility yet limited mechanical stability. In contrast, chemically crosslinked hydrogels boost structural integrity but may raise risks including residual crosslinkers, degradation byproducts, and chronic inflammation. Furthermore, the narrow intraocular space is highly sensitive to foreign−body reactions. Hydrogels intended for intraocular or intracapsular use require long−term functional safety evaluation beyond short−term cytocompatibility assessment, covering indicators such as corneal endothelium, iris, ciliary body, retina, optic nerve, IOL centration, posterior capsule contraction, and visual quality. Meanwhile, release behaviors of smart−responsive hydrogels suffer from insufficient *in vivo* predictability. Individual variations exist in tear volume, pH, reactive oxygen species levels, inflammatory factors, enzyme expression, and pathogen burden among patients. Excessively rapid release may trigger local toxicity or insufficient therapeutic persistence, whereas overly slow release could miss critical time windows for infection and inflammation control (10, 22). Hence, future research should establish release evaluation systems, long−term pharmacokinetic models, and large−animal validation platforms that better mimic real−world ocular conditions, rather than inferring clinical efficacy merely from *in−vitro* PBS release profiles or short−term small−animal experiments.

Future ocular hydrogel design shall shift from “material availability” to “disease−stage matching” and “clinical−scenario translatability”. For DED, composite eye−drop systems with adhesion, lubrication, antioxidation, mucin restoration and neuromodulation capacities need development. For corneal injuries, repair hydrogels featuring basement−membrane biomimicry, ECM−mimetic properties, low scarring and high transparency should be constructed to reduce reliance on sutures and donor corneas. For IK, programmable hydrogels that exert sequential functions across infection, inflammation, proliferation and remodeling phases require exploration. For uveitis, efforts should enhance non−invasive delivery efficiency of macromolecular biologics and strengthen synergy among anti−inflammatory, antioxidant and immunomodulatory effects. For glaucoma, stable long−acting IOP−lowering platforms built around drug−loaded contact lenses, ocular surface patches and minimally invasive implantable systems are demanded. For PCO prevention, combinations of controlled drug release, antifouling surfaces, spatial isolation and functionalized IOL structures can achieve one−step intraoperative intervention and long−term prophylaxis. From a materials science perspective, technologies including dynamic covalent chemistry, supramolecular self−assembly, Janus interfaces, nanozymes, cytokine adsorption and three−dimensional printing will further improve structural controllability and functional integration of hydrogels. From a clinical−translation standpoint, future priorities include sterile manufacturing, batch−to−batch stability, repeatable administration, removability, patient comfort and quality control criteria. In summary, hydrogels enable the transition of AS disease therapy from conventional eye drops with low bioavailability toward local immune−microenvironment−remodeling platforms. With interdisciplinary advances across materials science, ophthalmic pathology, drug delivery and clinical engineering, next−generation ocular hydrogels are expected to evolve into novel therapeutic systems with standardization potential, scalable production and clinical−translation prospects.

## References

[1] Subhash NE Prabhu M Hazarika M S S Bhandary SV Guru BR . Advances in the management of ocular anterior segment diseases using biomaterials-based drug delivery systems. J Biomater Appl. (2026) 40:666–89. doi: 10.1177/08853282251369229 40811684 PMC12638462

[2] Onugwu AL Nwagwu CS Onugwu OS Echezona AC Agbo CP Ihim SA . Nanotechnology based drug delivery systems for the treatment of anterior segment eye diseases. J Controlled Release. (2023) 354:465–88. doi: 10.1016/j.jconrel.2023.01.018 36642250

[3] Ahmed B Jaiswal S Naryal S Shah RM Alany RG Kaur IP . In situ gelling systems for ocular drug delivery. J Controlled Release. (2024) 371:67–84. doi: 10.1016/j.jconrel.2024.05.031 38768662

[4] Akbari E Imani R Shokrollahi P Jarchizadeh R Heidari Keshel S . Hydrogel-based formulations for drug delivery to the anterior segment of the eye. J Drug Del Sci Technol. (2023) 81:104250. doi: 10.1016/j.jddst.2023.104250 38826717

[5] Cooper RC Yang H . Hydrogel-based ocular drug delivery systems: Emerging fabrication strategies, applications, and bench-to-bedside manufacturing considerations. J Controlled Release. (2019) 306:29–39. doi: 10.1016/j.jconrel.2019.05.034 31128143 PMC6629478

[6] Wang X Li F Liu X Zhang H . Applications and recent developments of hydrogels in ophthalmology. ACS Biomat Sci Eng. (2023) 9:5968–84. doi: 10.1021/acsbiomaterials.3c00672 37906698

[7] Bourne R Steinmetz JD Flaxman S Briant PS Taylor HR Resnikoff S . Trends in prevalence of blindness and distance and near vision impairment over 30 years: An analysis for the global burden of disease study. Lancet Global Health. (2021) 9:e130–43. doi: 10.1016/S2214-109X(20)30425-3 33275950 PMC7820390

[8] Burton MJ Ramke J Marques AP Bourne RRA Congdon N Jones I . The Lancet Global Health Commission on global eye health: Vision beyond 2020. Lancet Global Health. (2021) 9:e489–551. doi: 10.1016/S2214-109X(20)30488-5 33607016 PMC7966694

[9] Flaxman SR Bourne RRA Resnikoff S Ackland P Braithwaite T Cicinelli MV . Global causes of blindness and distance vision impairment 1990-2020: A systematic review and meta-analysis. Lancet Global Health. (2017) 5:e1221–34. doi: 10.1016/S2214-109X(17)30393-5 29032195

[10] Wang X Luan F Yue H Song C Wang S Feng J . Recent advances of smart materials for ocular drug delivery. Adv Drug Del Rev. (2023) 200:115006. doi: 10.1016/j.addr.2023.115006 37451500

[11] Kim TY Lee GH Mun J Cheong S Choi I Kim H . Smart contact lens systems for ocular drug delivery and therapy. Adv Drug Del Rev. (2023) 196:114817. doi: 10.1016/j.addr.2023.114817 37004938

[12] Hakim FE Farooq AV . Dry eye disease: An update in 2022. JAMA. (2022) 327:478–9. doi: 10.1001/jama.2021.19963 35103781

[13] Sheppard J Shen Lee B Periman LM . Dry eye disease: Identification and therapeutic strategies for primary care clinicians and clinical specialists. Ann Med. (2023) 55:241–52. doi: 10.1080/07853890.2022.2157477 36576348 PMC9809411

[14] Luo LJ Nguyen DD Huang CC Lai JY . Therapeutic hydrogel sheets programmed with multistage drug delivery for effective treatment of corneal abrasion. Chem Eng J. (2022) 429:132409. doi: 10.1016/j.cej.2021.132409 38826717

[15] Li M Wei R Liu C Fang H Yang W Wang Y . A “T.E.S.T.” hydrogel bioadhesive assisted by corneal cross-linking for in situ sutureless corneal repair. Bioact Mater. (2023) 25:333–46. doi: 10.1016/j.bioactmat.2023.02.006 36844364 PMC9946819

[16] Tang Q Lu B He J Chen X Fu Q Han H . Exosomes-loaded thermosensitive hydrogels for corneal epithelium and stroma regeneration. Biomaterials. (2022) 280:121320. doi: 10.1016/j.biomaterials.2021.121320 34923312

[17] He B Wang J Xie M Xu M Zhang Y Hao H . 3d printed biomimetic epithelium/stroma bilayer hydrogel implant for corneal regeneration. Bioact Mater. (2022) 17:234–47. doi: 10.1016/j.bioactmat.2022.01.034 35386466 PMC8965162

[18] Borouman S Sigaroodi F Ahmadi Tafti SM Khoshmaram K Soleimani M Khani M-M . Ecm-based bioadhesive hydrogel for sutureless repair of deep anterior corneal defects. Biomater Sci. (2024) 12:2356–68. doi: 10.1039/D4BM00129J 38497791

[19] Wu KY Qian SY Faucher A Tran SD . Advancements in hydrogels for corneal healing and tissue engineering. Gels. (2024) 10:662. doi: 10.3390/gels10100662 39451315 PMC11507397

[20] Ting DSJ Ho CS Deshmukh R Said DG Dua HS . Infectious keratitis: An update on epidemiology, causative microorganisms, risk factors, and antimicrobial resistance. Eye. (2021) 35:1084–101. doi: 10.1038/s41433-020-01339-3 33414529 PMC8102486

[21] Rhee MK Ahmad S Amescua G Cheung AY Choi DS Jhanji V . Bacterial keratitis preferred practice pattern®. Ophthalmology. (2024) 131:P87–P133. doi: 10.1016/j.ophtha.2023.12.035 38826717

[22] Fan Y Chen F Yuan W Sun Y Li J Li Y . Immunoregulatory cryogel-based contact lenses for bacterial keratitis prevention and treatment. Cell Rep Phys Sci. (2024) 5. doi: 10.1016/j.xcrp.2024.102179 38826717

[23] Ruan M Wang R He Y . Novel drug delivery systems for the management of fungal keratitis. J Ocular Pharmacol Ther. (2024) 40:160–72. doi: 10.1089/jop.2023.0161 38394222

[24] Daniel Raj Ponniah LR Ranilakshmi V Anandan H Caroline J Arulanandham A . Novel drug-repository contact lens for prolonging the antimicrobial-cornea interaction for bacterial keratitis treatment: Randomised controlled trial results. BMJ Open Ophthalmol. (2022) 7:e001093. doi: 10.1136/bmjophth-2022-001093

[25] Jayaram H Kolko M Friedman DS Gazzard G . Glaucoma: now and beyond. Lancet. (2023) 402:1788–801. doi: 10.1016/S0140-6736(23)01289-8 37742700

[26] Tham Y-C Li X Wong TY Quigley HA Aung T Cheng C-Y . Global prevalence of glaucoma and projections of glaucoma burden through 2040: A systematic review and meta-analysis. Ophthalmology. (2014) 121:2081–90. doi: 10.1016/j.ophtha.2014.05.013 24974815

[27] Fea AM Vallino V Cossu M Marica V Novarese C Reibaldi M . Drug delivery systems for glaucoma: A narrative review. Pharmaceuticals. (2024) 17:1163. doi: 10.3390/ph17091163 39338326 PMC11435076

[28] Maghsoudlou P Epps SJ Guly CM Dick AD . Uveitis in adults: A review. JAMA. (2025) 334:419–34. doi: 10.1001/jama.2025.4358 40434762

[29] Annala A Ilochonwu BC Wilbie D Sadeghi A Hennink WE Vermonden T . Self-healing thermosensitive hydrogel for sustained release of dexamethasone for ocular therapy. ACS Polymers Au. (2023) 3:118–31. doi: 10.1021/acspolymersau.2c00038 36785837 PMC9912331

[30] Wu X Yang L Chen Q Ran R Cao J Zhang M . Microenvironment-responsive hydrogels with drug-loaded microspheres for sustained dexamethasone acetate release and experimental autoimmune uveitis suppression. J Mater Chem B. (2026) 14:1615–29. doi: 10.1039/D5TB02106E 41532726

[31] Wormstone IM Wormstone YM Smith AJO Eldred JA . Posterior capsule opacification: What's in the bag? Prog Retinal Eye Res. (2021) 82:100905. doi: 10.1016/j.preteyeres.2020.100905 32977000

[32] Jumelle C Gholizadeh S Annabi N Dana R . Advances and limitations of drug delivery systems formulated as eye drops. J Controlled Release. (2020) 321:1–22. doi: 10.1016/j.jconrel.2020.01.057 32027938 PMC7170772

[33] So YH Mishra D Gite S Sonawane R Waite D Shaikh R . Emerging trends in long-acting sustained drug delivery for glaucoma management. Drug Del Trans Res. (2025) 15:1907–34. doi: 10.1007/s13346-024-01779-4 39786666 PMC12037438

[34] Belamkar A Harris A Zukerman R Siesky B Oddone F Verticchio Vercellin A . Sustained release glaucoma therapies: Novel modalities for overcoming key treatment barriers associated with topical medications. Ann Med. (2022) 54:343–58. doi: 10.1080/07853890.2021.1955146 35076329 PMC8794062

[35] Li X Liang C Guo Y Su J Chen X Macgregor RB . Clinical translation of long-acting drug delivery systems for posterior capsule opacification prophylaxis. Pharmaceutics. (2023) 15:1235. doi: 10.3390/pharmaceutics15041235 37111720 PMC10143098

[36] Qin C Wen S Fei F Han Y Wang H Chen H . Nir-triggered thermosensitive polymer brush coating modified intraocular lens for smart prevention of posterior capsular opacification. J Nanobiotech. (2023) 21:323. doi: 10.1186/s12951-023-02055-2 37679734 PMC10483730

[37] Ying-Yan M Meng L Jin-Da W Kai-Jie W Jing-Shang Z Shu-Ying C . Nir-triggered drug delivery system for chemo-photothermal therapy of posterior capsule opacification. J Controlled Release. (2021) 339:391–402. doi: 10.1016/j.jconrel.2021.09.030 34563593

[38] Zhang X Wang J Xu J Xu W Zhang Y Luo C . Prophylaxis of posterior capsule opacification through autophagy activation with indomethacin-eluting intraocular lens. Bioact Mater. (2023) 23:539–50. doi: 10.1016/j.bioactmat.2022.11.024 36514385 PMC9729928

[39] Xiang Y Jin R Zhang Y Li K Liu G Song X . Foldable glistening-free acrylic intraocular lens biomaterials with dual-side heterogeneous surface modification for postoperative endophthalmitis and posterior capsule opacification prophylaxis. Biomacromolecules. (2021) 22:3510–21. doi: 10.1021/acs.biomac.1c00582 34288655

[40] Fang G Yang X Wang Q Zhang A Tang B . Hydrogels-based ophthalmic drug delivery systems for treatment of ocular diseases. Mater Sci Eng C. (2021) 127:112212. doi: 10.1016/j.msec.2021.112212 34225864

[41] Sabbagh F Zargarian SS Kosik-Kozioł A Nakielski P Pierini F . Hydrogel-based ocular drug delivery systems. J Mater Chem B. (2025) 13:14982–5006. doi: 10.1039/D5TB01575H 41183353

[42] Li S Chen L Fu Y . Nanotechnology-based ocular drug delivery systems: Recent advances and future prospects. J Nanobiotech. (2023) 21:232. doi: 10.1186/s12951-023-01992-2 37480102 PMC10362606

[43] Yu H Yu X Huang Y Yu T Lan H Zhang Q . Engineering biocompatible carbon dots nano-enzymes hydrogel for efficient antioxidative and anti-inflammatory treatment of dry eye disease. J Controlled Release. (2025) 381:113490. doi: 10.1016/j.jconrel.2025.01.081 39884436

[44] Ding X Ben-Shlomo G Que L . Soft contact lens with embedded microtubes for sustained and self-adaptive drug delivery for glaucoma treatment. ACS Appl Mat Interfaces. (2020) 12:45789–95. doi: 10.1021/acsami.0c12667 32960561

[45] Manjeri A George SD . Hydrogel-embedded polydimethylsiloxane contact lens for ocular drug delivery. ACS Appl Bio Mater. (2024) 7:7324–31. doi: 10.1021/acsabm.4c00975 39425674 PMC11577423

[46] Wang F Song Y Huang J Wu B Wang Y Pang Y . Lollipop-inspired multilayered drug delivery hydrogel for dual effective, long-term, and nir-defined glaucoma treatment. Macromol Biosci. (2021) 21:2100202. doi: 10.1002/mabi.202100202 34405963

[47] Li Q Cao Y Wang P . Recent advances in hydrogels for the diagnosis and treatment of dry eye disease. Gels. (2022) 8:816. doi: 10.3390/gels8120816 36547340 PMC9778550

[48] van Zyl T Yan W McAdams AM Monavarfeshani A Hageman GS Sanes JR . Cell atlas of the human ocular anterior segment: Tissue-specific and shared cell types. Proc Natl Acad Sci. (2022) 119:e2200914119. doi: 10.1073/pnas.2200914119 35858321 PMC9303934

[49] Ahsanuddin S Wu AY . Single-cell transcriptomics of the ocular anterior segment: A comprehensive review. Eye. (2023) 37:3334–50. doi: 10.1038/s41433-023-02539-3 37138096 PMC10156079

[50] de Paiva CS St Leger AJ Caspi RR . Mucosal immunology of the ocular surface. Mucosal Immunol. (2022) 15:1143–57. doi: 10.1038/s41385-022-00551-6 36002743 PMC9400566

[51] Weinreb RN Leung CKS Crowston JG Medeiros FA Friedman DS Wiggs JL . Primary open-angle glaucoma. Nat Rev Dis Primers. (2016) 2:16067. doi: 10.1038/nrdp.2016.67 27654570

[52] Cheng Y-H Huang H-P Chen H-H . Mucoadhesive phenylboronic acid-grafted carboxymethyl cellulose hydrogels containing glutathione for treatment of corneal epithelial cells exposed to benzalkonium chloride. Coll Surfaces B Biointerfaces. (2024) 238:113884. doi: 10.1016/j.colsurfb.2024.113884 38565006

[53] Li M Guo H Wang B Han Z Wu S Liu J . The single-cell transcriptomic atlas and Rora-mediated 3d epigenomic remodeling in driving corneal epithelial differentiation. Nat Commun. (2024) 15:256. doi: 10.1038/s41467-023-44471-w 38177186 PMC10766623

[54] Matsuzawa M Ando T Fukase S Kimura M Kume Y Ide T . The protective role of conjunctival goblet cell mucin sialylation. Nat Commun. (2023) 14:1417. doi: 10.1038/s41467-023-37101-y 36932081 PMC10023771

[55] Thomson BR Liu P Onay T Du J Tompson SW Misener S . Cellular crosstalk regulates the aqueous humor outflow pathway and provides new targets for glaucoma therapies. Nat Commun. (2021) 12:6072. doi: 10.1038/s41467-021-26346-0 34663817 PMC8523664

[56] Ouyang W Wang S Yan D Wu J Zhang Y Li W . The cGAS-STING pathway-dependent sensing of mitochondrial DNA mediates ocular surface inflammation. Signal Transduct Tgt Ther. (2023) 8:371. doi: 10.1038/s41392-023-01624-z 37735446 PMC10514335

[57] Minns MS Liboro K Lima TS Abbondante S Miller BA Marshall ME . NLRP3 selectively drives IL-1β secretion by Pseudomonas aeruginosa infected neutrophils and regulates corneal disease severity. Nat Commun. (2023) 14:5832. doi: 10.1038/s41467-023-41391-7 37730693 PMC10511713

[58] Wang JD Zhang JS Li XX Wang KJ Li M Mao YY . Knockout of TGF-β receptor II by CRISPR/Cas9 delays mesenchymal transition of lens epithelium and posterior capsule opacification. Int J Biol Macromol. (2024) 259:129290. doi: 10.1016/j.ijbiomac.2024.129290 38199534

[59] Cabrera-Aguas M Khoo P Watson SL . Infectious keratitis: a review. Clin Exp Ophthalmol. (2022) 50:543–62. doi: 10.1111/ceo.14113 35610943 PMC9542356

[60] Garaszczuk IK Mousavi M Cervino Exposito A Bartuzel MM Montes-Micó R Iskander DR . Evaluating tear clearance rate with optical coherence tomography. Contact Lens Anterior Eye. (2018) 41:54–9. doi: 10.1016/j.clae.2017.08.004 28847465

[61] Ranjith K Nagapriya B Shivaji S . Polymicrobial biofilms of ocular bacteria and fungi on ex vivo human corneas. Sci Rep. (2022) 12:11606. doi: 10.1038/s41598-022-15809-z 35803992 PMC9270462

[62] Medeiros FA Walters TR Kolko M Coote M Bejanian M Goodkin ML . Phase 3, randomized, 20-month study of bimatoprost implant in open-angle glaucoma and ocular hypertension (Artemis 1). Ophthalmology. (2020) 127:1627–41. doi: 10.1016/j.ophtha.2020.06.018 32544560

[63] Ou L Wu Z Hu X Huang J Yi Z Gong Z . A tissue-adhesive F127 hydrogel delivers antioxidative copper-selenide nanoparticles for the treatment of dry eye disease. Acta Biomater. (2024) 175:353–68. doi: 10.1016/j.actbio.2023.12.021 38110136

[64] Chen Z Yang M Wang Q Bai J McAlinden C Skiadaresi E . Hydrogel eye drops as a non-invasive drug carrier for topical enhanced adalimumab permeation and highly efficient uveitis treatment. Carbohydr Polym. (2021) 253:117216. doi: 10.1016/j.carbpol.2020.117216 33278980

[65] Liu X Chen Z Bai J Li X Chen X Li Z . Multifunctional hydrogel eye drops for synergistic treatment of ocular inflammatory disease. ACS Nano. (2023) 17:25377–90. doi: 10.1021/acsnano.3c08869 37890030

[66] Huang J Jiang T Qie J Cheng X Wang Y Ye Y . Biologically inspired bioactive hydrogels for scarless corneal repair. Sci Adv. (2024) 10:eadt1643. doi: 10.1126/sciadv.adt1643 39693435 PMC11654680

[67] Shen X Li S Zhao X Han J Chen J Rao Z . Dual-crosslinked regenerative hydrogel for sutureless long-term repair of corneal defect. Bioact Mater. (2023) 20:434–48. doi: 10.1016/j.bioactmat.2022.06.006 35800407 PMC9234351

[68] Meng S Hu H Qiao Y Wang F Zhang BN Sun D . A versatile hydrogel with antibacterial and sequential drug-releasing capability for the programmable healing of infectious keratitis. ACS Nano. (2023) 17:24055–69. doi: 10.1021/acsnano.3c09034 38044579

[69] Geng W Lu Y Xu H Song Y Liu S Liu X . Engineering multifunctional chitosan-based hydrogels for integrated antimicrobial therapy and endogenous stem cell-driven tissue repair in infectious keratitis. Bioact Mater. (2026) 62:569–86. doi: 10.1016/j.bioactmat.2026.03.026 42293731 PMC13261646

[70] Shajari G Erfan-Niya H Fathi M Amiryaghoubi N . In situ forming hydrogels based on modified gellan gum/chitosan for ocular drug delivery of timolol maleate. Int J Biol Macromol. (2024) 278:135071. doi: 10.1016/j.ijbiomac.2024.135071 39187113

[71] Yin C Zhang Y Fan C Zheng J Yang Y Zhang Y . Injectable and pH-responsive metformin-loaded hydrogel for active inhibition of posterior capsular opacification. ACS Appl Mat Interfaces. (2024) 16:59880–94. doi: 10.1021/acsami.4c13318 39437316

[72] Yang CJ Huang WL Yang Y Kuan CH Tseng CL Wang TW . Zwitterionic modified and freeze-thaw reinforced foldable hydrogel as intraocular lens for posterior capsule opacification prevention. Biomaterials. (2024) 309:122593. doi: 10.1016/j.biomaterials.2024.122593 38713971

[73] Zöller K To D Bernkop-Schnürch A . Biomedical applications of functional hydrogels: Innovative developments, relevant clinical trials and advanced products. Biomaterials. (2025) 312:122718. doi: 10.1016/j.biomaterials.2024.122718 39084097

[74] Han J Shu H Zhang L Huang S . Latest advances in hydrogel therapy for ocular diseases. Polymer. (2024) 306:127207. doi: 10.1016/j.polymer.2024.127207 38826717

[75] Pham DT Nguyen NY Jeencham R Tiyaboonchai W . Natural biomaterials for contact lens-based ophthalmic drug delivery systems. J Controlled Release. (2025) 387:114171. doi: 10.1016/j.jconrel.2025.114171 40882773

[76] Meng C Nan W Wu M Guo Q Zhang W Wu H . Functionalized metal–organic framework-based photosensitive hydrogel eye drops for inhibition of corneal neovascularization and promotion of epithelial repair. J Nanobiotech. (2026) 24:307. doi: 10.1186/s12951-026-04314-4 41923125 PMC13047812

[77] Pan M Ren Z Ma X Chen L Lv G Liu X . A biomimetic peptide–drug supramolecular hydrogel as eyedrops enables controlled release of ophthalmic drugs. Acta Biomater. (2023) 167:195–204. doi: 10.1016/j.actbio.2023.06.036 37392932

[78] Zhang W Zhao M Chu D Chen H Cui B Ning Q . Dual-ROS-scavenging and dual-lingering nanozyme-based eye drops alleviate dry eye disease. J Nanobiotech. (2024) 22:229. doi: 10.1186/s12951-024-02499-0 38720321 PMC11077849

[79] Wei W Cao H Shen D Sun X Jia Z Zhang M . Antioxidant carbon dots nanozyme loaded in thermosensitive in situ hydrogel system for efficient dry eye disease treatment. Int J Nanomed. (2024) 19:4045–60. doi: 10.2147/IJN.S456613 38736656 PMC11088389

[80] Wang X Zhou W Xue H Xue W Gao G Zhu B . Advanced corneal hydrogels: From passive replacement to active regeneration and intelligent interaction. Adv Sci. (2026) 13:e22362. doi: 10.1002/advs.202522362 41736701 PMC13045501

[81] Wu K Lu S Hu Y Guo M Wang G Wei D . Enzyme-mimetic hydrogel balancing antibacterial activity and cytoprotection for corneal regeneration in bacterial keratitis. ACS Nano. (2026) 20:7300–20. doi: 10.1021/acsnano.5c22118 41689540

[82] Dang M Shoichet MS . Long-acting ocular injectables: Are we looking in the right direction? Adv Sci. (2024) 11:e2306463. doi: 10.1002/advs.202306463 38018313 PMC10885661

[83] Qin C Fei F Wei Y Han Y Hu D Lin Q . Thermo-sensitive poloxamer based antibacterial anti-inflammatory and photothermal conductive multifunctional hydrogel as injectable, in situ curable and adjustable intraocular lens. Bioact Mater. (2024) 41:30–45. doi: 10.1016/j.bioactmat.2024.07.005 39101029 PMC11292259

[84] Ilochonwu BC van der Lugt SA Annala A Di Marco G Sampon T Siepmann J . Thermo-responsive Diels-Alder stabilized hydrogels for ocular drug delivery of a corticosteroid and an anti-VEGF Fab fragment. J Controlled Release. (2023) 361:334–49. doi: 10.1016/j.jconrel.2023.07.052 37532147

[85] Sun R Zhang J Chen X Deng Y Gou J Yin T . An adaptive drug-releasing contact lens for personalized treatment of ocular infections and injuries. J Controlled Release. (2024) 369:114–27. doi: 10.1016/j.jconrel.2024.03.040 38521167

[86] Fan K Yang D Zhu X Zheng L Han Y Lin J . High-efficiency antioxidant ROS-responsive thermosensitive hydrogel encapsulated fenofibrate for the treatment of corneal neovascularization. J Controlled Release. (2025) 382:113650. doi: 10.1016/j.jconrel.2025.113650 40120688

[87] Liu Y Ren H Wu Z Wu Y Zhou X Ji D . Advances in the application of smart materials in the treatment of ophthalmic diseases. Biomaterials. (2025) 321:123316. doi: 10.1016/j.biomaterials.2025.123316 40194319

[88] Li Y Wang X Wu M Ren J Wang Y Wen C . Micelle-integrated hydrogel combined with pH-response boosts eye burns therapy by inhibiting neovascularization, regulating inflammation and bacteriostasis. Biomater Adv. (2026) 183:214732. doi: 10.1016/j.bioadv.2026.214732 41643408

[89] Wang JJ Liu XX Zhu CC Wang TZ Wang SY Liu Y . Improving ocular bioavailability of hydrophilic drugs through dynamic covalent complexation. J Controlled Release. (2023) 355:395–405. doi: 10.1016/j.jconrel.2023.01.081 36739907

[90] Li Y Wang Z . Biomaterials for corneal regeneration. Adv Sci. (2025) 12:2408021. doi: 10.1002/advs.202408021 39739318 PMC11809424

[91] Chen F Han U Wungcharoen T Seo YA Le P Jiang L . Bio-orthogonal crosslinking and hyaluronan facilitate transparent healing after treatment of deep corneal injuries with in situ-forming hydrogels. NPJ Regen Med. (2025) 10:8. doi: 10.1038/s41536-024-00385-9 39905045 PMC11794660

[92] Dong L Fan Z Fang B Zhao X Yao H Cai G . Oriented cellulose hydrogel: Directed tissue regeneration for reducing corneal leukoplakia and managing fungal corneal ulcers. Bioact Mater. (2024) 41:15–29. doi: 10.1016/j.bioactmat.2024.07.008 39101028 PMC11292264

[93] Pan X Wang Z Wu X Guo C Yang L Liu H . ROS scavenging and corneal epithelial wound healing by a self-crosslinked tissue-adhesive hydrogel based-on dual-functionalized hyaluronic acid. Int J Biol Macromol. (2025) 293:139200. doi: 10.1016/j.ijbiomac.2024.139200 39730051

[94] Qie J Wang Y Huang J He X Ye Y Yao K . A customized Janus hydrogel with both anti‐fibrosis and anti‐infection properties for suture‐free and care‐free corneal injury repair. Adv Funct Mater. (2025) 36:e13261. doi: 10.1002/adfm.202513261 41531421

[95] Yang M Chen X Chen Z Zhao N Zeng Z Huang X . Thermoresponsive antioxidant metal-free carbon nanodot hydrogel: An effective therapeutic approach for ocular surface disease. Sci Adv. (2025) 11:eadt8775. doi: 10.1126/sciadv.adt8775 40712015 PMC12292845

[96] Wang Y Dong Z Yao H Li Y Cui D Cao B . A dual-pronged strategy for bacterial keratitis: ROS-responsive hydrogel eye drops enabling potent deep-tissue antibacterial action and NETs modulation. Bioact Mater. (2026) 64:16–33. doi: 10.1016/j.bioactmat.2026.04.036 42093835 PMC13141534

